# Convention of American Medical Editors

**Published:** 1878-07

**Authors:** 


### Convention of American Medical Editors.

The Tenth Annual Convention of the Association of Medical Editors was held Monday evening, June 3d, at the Tifft House, Dr. J. B. Gray, Superintendent of the New York State Asylum at

Utica, in the chair. The following members were present: Dr. John P. Gray, Utica, *American Journal of Insanity;* Dr. William Brodie, *Michigan Medical News;* Dr. Theophilus Parvin, *American Practitioner;* Dr. C. Henri Leonard, *New Preparations;* Dr. J. J. Mulheron, Dr. E. S. Dunster, *Michigan Medical News;* Dr. Laertus Connor, *Detroit Lancet;* Dr. O. Cowling, *Louisville Medical News;* Drs. N. S. and F. H. Davis, late of the *Chicago Medical Examiner;* Dr. T. F. Rumbold, *St. Louis Medical and Surgical Examiner;* Drs. J. F. Miner, E. N. Brush and W. W. Miner, *Buffalo Medical and Surgical Journal.* A number of physicians unconnected with medical journals were also present as guests. The annual paper was read by the President, Dr. Gray, who took for his subject the question of the laws of England and the State of New York for committing, detaining and discharging the insane from confinement.

The thanks of the Association were voted to Dr. Gray for his able and interesting paper. It was then opened for discussion.

Dr. Davis of Chicago said he was sorry the laws of other States had not been brought into the paper so that the Association might realize their defects. In Illinois, for instance, no insane person could be placed in confinement without first undergoing the shame of a public trial by jury. The injury to many patients was inconceivable.

Dr. Foster Pratt of Kalamazoo spoke of the laws of Michigan. In that State, he said, patients were in more danger from the courts than from the statutes. The Michigan laws were to a great degree similar to those of New York. He believed that relatives ought to have a right to take care of their insane as well as their other sick ones. If they neglected their duty, punish them; if they abused their power, punish them.

Dr. Gray spoke briefly of the terrible consequences arising from the Illinois law of bringing all sorts of patients, men and women alike, into a public court room.

Drs. Brush and Dunster were appointed a nominating committee, and upon their recommendation the following officers were elected for the ensuing year:

President, Dr. William Brodie, Detroit; Vice-President, Dr. J. F. Miner, Buffalo; Secretary, F. H. Davis, Chicago.

The meeting then adjourned.

